# Plasma Carboxyl-Metabolome Is Associated with Average Daily Gain Divergence in Beef Steers

**DOI:** 10.3390/ani11010067

**Published:** 2021-01-01

**Authors:** Ibukun Ogunade, Adeoye Oyebade, Bremansu Osa-Andrews, Sunday Peters

**Affiliations:** 1 Division of Animal and Nutritional Science, West Virginia University, Morgantown, WV 26506, USA; bremansu.osaandrews@mail.wvu.edu; 2 Department of Animal Sciences, University of Florida, Gainesville, FL 32611, USA; adeoye.oyebade@ufl.edu; 3 Department of Animal Science, Berry College, Mount Berry, GA 30149, USA; speters@Berry.edu

**Keywords:** fatty acids, metabolomics, plasma, enzymatic pathways

## Abstract

**Simple Summary:**

In this study, we analyzed the plasma carboxyl-metabolome in beef steers with divergent average daily gain (ADG). Several short chain fatty acids were greater in beef steers with greater ADG. Conversely, several long chain fatty acids were greater in beef steers with lower ADG. Pathway analysis of the differential metabolites revealed alterations in abundance/activities of enzymes involved in fatty acid metabolism in the liver. The results of this study demonstrated that beef steers with divergent ADG had altered plasma carboxyl-metabolome, which is possibly caused by altered abundances and/or activities of enzymes involved in fatty acid oxidation and biosynthesis.

**Abstract:**

We applied an untargeted metabolomics technique to analyze the plasma carboxyl-metabolome of beef steers with divergent average daily gain (ADG). Forty-eight newly weaned Angus crossbred beef steers were fed the same total mixed ration ad libitum for 42 days. On day 42, the steers were divided into two groups of lowest (LF: *n* = 8) and highest ADG (HF: *n* = 8), and blood samples were obtained from the two groups for plasma preparation. Relative quantification of carboxylic-acid-containing metabolites in the plasma samples was determined using a metabolomics technique based on chemical isotope labeling liquid chromatography mass spectrometry. Metabolites that differed (fold change (FC) ≥ 1.2 or ≤ 0.83 and FDR ≤ 0.05) between LF and HF were identified using a volcano plot. Metabolite set enrichment analysis (MSEA) of the differential metabolites was done to determine the metabolic pathways or enzymes that were potentially altered. In total, 328 metabolites were identified. Volcano plot analysis revealed 43 differentially abundant metabolites; several short chain fatty acids and ketone bodies had greater abundance in HF steers. Conversely, several long chain fatty acids were greater in LF steers. Five enzymatic pathways, such as fatty acyl CoA elongation and fatty-acid CoA ligase were altered based on MSEA. This study demonstrated that beef steers with divergent ADG had altered plasma carboxyl-metabolome, which is possibly caused by altered abundances and/or activities of enzymes involved in fatty acid oxidation and biosynthesis in the liver.

## 1. Introduction

Improving feed efficiency in cattle is crucial for reducing feed costs and environmental impact of animal production [1]. Thus, several studies have focused on understanding the biological mechanisms that cause differences in feed efficiency-related traits in ruminants. Previous studies applied metabolomics and metagenomics analysis of rumen microbiota as well as transcriptomics and proteomics analysis of liver to examine differences in average daily gain (ADG) and/or residual feed intake (RFI) in animals [2,3,4]; however, these studies involve invasive and time-consuming sample collection procedures. Due to the convenience and relatively non-invasive accessibility of blood samples, and the potential utility of blood metabolome as a functional read-out of overall metabolisms in the body [5], blood plasma has been the most widely examined sample type in bovine metabolomics studies [6].

High-performance chemical isotope labeling (CIL) liquid chromatography mass spectrometry (LC–MS)-based metabolomics technique is an important tool used to profile chemical-group-based metabolomes such as amine/phenol (metabolites containing amine/phenol groups) and carboxyl-metabolome (metabolites containing carboxylic acid groups) [7]. In our previous study, we applied a CIL/LC–MS-based metabolomics technique to examine differences in the plasma metabolites of beef steers divergent in ADG with a focus on those metabolites containing amine/phenol chemical groups [8]. However, previous studies that studied transcriptomics and proteomics analysis of liver tissues identified fatty acid metabolism as the most important metabolic pathway associated with feed efficiency-related traits in animals [2,4,9]. Thus, we hypothesized that beef steers divergent in ADG would have altered plasma carboxyl-metabolome profile. Therefore, this study applied CIL-LC-MS-based metabolomics to analyze carboxylic-acid-containing metabolites (carboxyl-metabolome), including fatty acid and their derivatives, in the plasma of beef steers divergent in ADG.

## 2. Materials and Methods

### 2.1. Animals, Feeding, and Growth Performance

All experimental animals were managed according to guidelines approved by the Institutional Animal Care and Use Committee of Kentucky State University (18-0001). Details about animals, feeding, and measurements of dry matter intake and average daily gain have been reported previously [8]. Briefly, 48 Angus crossbred beef steers (21 d post-weaning; 210 ± 8.5 kg of body weight) were individually housed in slatted floor pens (2.44 × 14.63 m^2^) and fed ad libitum a 79% corn silage and 21% grain mix-based total mixed ration with free access to water for 42 d after a 21 d adaptation period (63 d total). The grain mix contained distillers’ grain, soybean meal, and limestone (CP = 14.5% and NE_g_ = 1.10 Mcal/kg). Daily feed offered and refused (as-fed) by each steer were recorded. Daily DM intake of each steer was determined by the difference between daily DM offered and daily DM refused. Body weights of the steers were also obtained on d 0 and 42 before morning feeding. Average daily gain (ADG) was calculated by dividing the total body weight gain during the 42 d period by the number of experimental days (42 days). Steers with the lowest (LF: *n* = 8) and highest ADG (HF: *n* = 8) were selected from the 48 steers.

On day 42, before the morning feeding, about 10 mL of blood was taken from the steers via the coccygeal vessels into tubes containing sodium heparin (Vacutainer, Becton Dickinson, Franklin Lakes, NJ, USA) and immediately placed on ice. Plasma samples were obtained within 15 min of collection by centrifugation at 2500× *g* for 20 min at 4 °C, and thereafter stored at −80 °C until untargeted metabolomics analysis.

### 2.2. CIL-LC/MS-Based Metabolomics

Relative quantification of carboxylic-acid-containing metabolites (carboxyl-metabolome) in plasma samples obtained from LF (*n* = 8) and HF steers (*n* = 8) was determined using a CIL/LC-MS-based metabolomics technique. One of the LF samples was damaged during processing; therefore, seven LF samples were analyzed. The CIL/LC-MS technique for carboxyl-metabolome applies an isotope labelling method based on the use of isotope-coded p-dimethylaminophenacyl bromide as a reagent, combined with LC-MS for high-performance metabolome analysis [7,10]. The metabolites were first extracted via methanol protein precipitation as previously described [7,11]. Detailed information on metabolite labelling, sample amount normalization using LC–ultraviolet quantification of the labeled metabolites, and quantification of the metabolites using an LC system (Agilent Technologies Inc., Palo Alto, CA, USA) connected to a Bruker Impact HD quadrupole time-of-flight MS have been previously reported [10,11,12].

#### Metabolite Data Processing and Identification

Raw LC-MS data (peak pairs) were processed using IsoMS Pro 1.0 [12]. Peak pairs whose mean (sample)/mean (blank) was ≤4.0 and/or with no data present in at least 80% of the samples were removed. A final metabolite-intensity table was generated using IsoMS-Quant [13]. Metabolite identification was done at 2 tier levels using IsoMS Pro software and database (Nova Medical Testing Inc., Edmonton, AB, Canada). The first-tier identification was done based on accurate mass and retention time search against labeled standard metabolite library, which is composed of 187 unique human endogenous carboxylic-acid-containing metabolites [7]. The second-tier identification was based on accurate mass and predicted retention time matches [14] against the Linked Identity Library containing metabolites related to metabolic pathway in KEGG database [7].

### 2.3. Statistical and Data analysis

The carboxyl-metabolome data were imported into Metaboanalyst 4.0 software (https://www.metaboanalyst.ca/) for statistical analysis [15]. The data were first normalized by median, log-transformed, and auto-scaled prior to statistical testing. A principal component analysis scores plot was used to visualize the difference between the LF and HF. A volcano plot was constructed using fold change (FC) in each metabolite against Benjamini-Hochberg false discovery rate (FDR) set to *p* ≤ 0.05. Relative to LF, metabolites with FC ≥ 1.2 or ≤ 0.83 and FDR ≤ 0.05 were considered differentially increased or decreased, respectively. Metabolite set enrichment analysis (MSEA) of the differentially abundant metabolites was performed to determine the metabolic pathways or enzymes that were potentially altered. The predicted metabolite library was set as the chosen metabolite library; this library contains 912 metabolic sets that are predicted to be changed in the case of dysfunctional enzymes using a genome-scale network model of human metabolism [15]. Pathways with *p* ≤ 0.10 were considered different between LF and HF steers.

## 3. Results

The results of the growth performance of LF and HF steers have been previously reported [8]. Briefly, the average initial body weight (229 vs. 225 kg; SE = 5.21) and average daily DM intake (6.08 vs. 6.04 kg; SE = 0.23) of the LF and HF beef steers were similar (*p* > 0.05). The final body weight (274 vs. 293 kg; SE = 2.89) and ADG (1.09 vs. 1.63; SE = 0.07) were lower (*p* = 0.01) for LF compared with HF steers.

**Figure 1:**
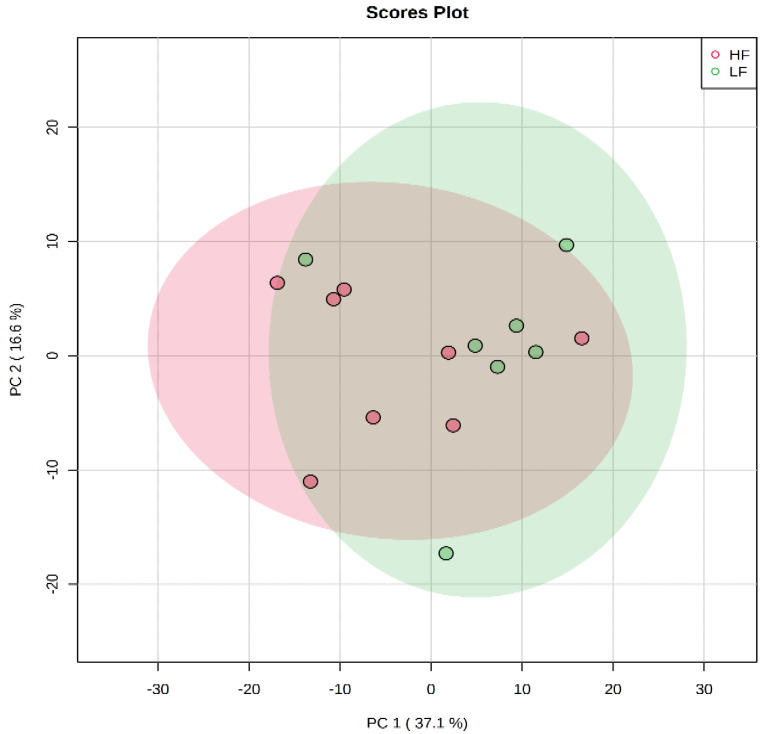
Principal component analysis scores plot of carboxyl-metabolome of LF and HF steers. LF = beef steers with lowest average daily gain; HF = beef steers with highest average daily gain.

**Figure 2:**
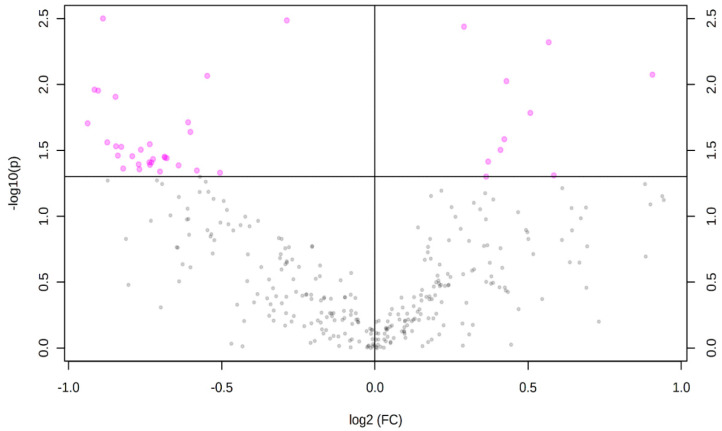
Volcano plot showing the differentially abundant carboxylic-acid-containing plasma metabolites (FC ≥ 1.2 or ≤ 0.83; *p* ≤ 0.05) between LF and HF steers. LF = beef steers with lowest average daily gain; HF = beef steers with highest average daily gain. Purple dots represent the differentially abundant metabolites.

A total number of 328 carboxylic-acid-containing metabolites were detected and identified in the plasma samples of LF and HF steers (Supplementary Table S1). Principal component analysis plot showed no separation between the plasma carboxyl-metabolome of the LF and HF steers (Figure 1). However, results of the volcano plot analysis revealed 43 differentially abundant (FC ≥ 1.2 or ≤ 0.83 and FDR ≤ 0.05) metabolites (Figure 2); the relative abundance of 11 metabolites including acetate, 3-hydroxybutyrate, 3-isohydroxybutyrate, butanoic acid, hydroxyisovalerate, leukotriene B4, and 11-hydroxy-14,15-epoxy-5Z,8Z,12E-eicosatrienoic acid (11H-14,15-EETA) were greater in HF steers, while the relative abundance 32 metabolites including retinoic acid and 31 long chain fatty acid (LCFA) and their derivatives such as oleic acid, linoleic acid, octadecatrienoic acid, 6-amino-2-oxohexanoate, arachidonic acid, eisosatrienoic acid, eisosadienoic acid, 18-oxooleate, myristic acid, pentadecylic acid, 9,10,18-trihydroxystearate, and 9,10-epoxy-18-hydroxystearate were greater in LF steers (Table 1).

**Figure 3:**
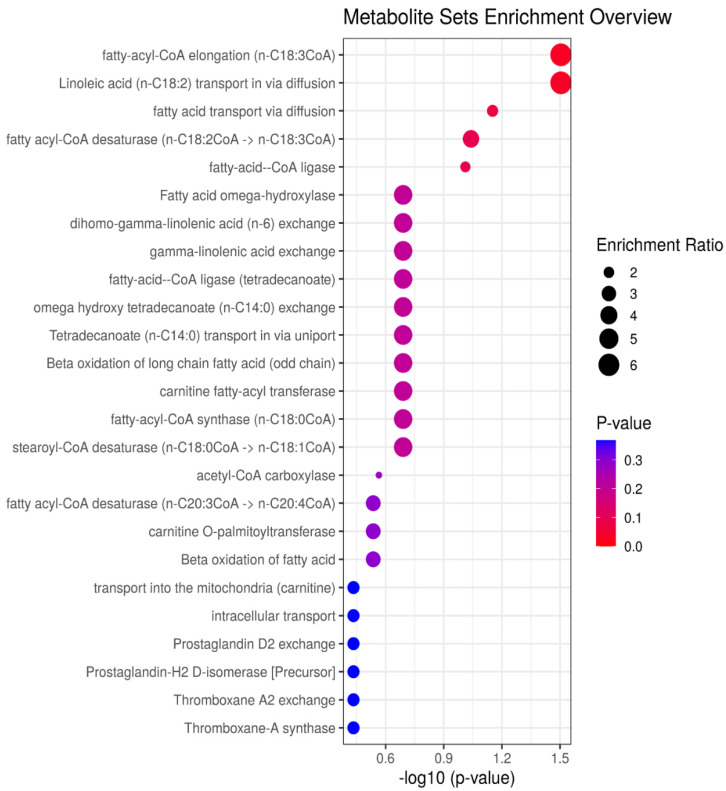
Results of the metabolite set enrichment analysis. Fatty acyl coA elongation, *p* = 0.03; linoleic acid (n-C18:2) transport in via diffusion, *p* = 0.03; fatty acid transport via diffusion, *p* = 0.07; fatty acyl-CoA desaturase, *p* = 0.09; fatty-acid-CoA ligase, *p* = 0.09.

Figure 3 displays the results of the MSEA, identifying five enzymatic pathways as being significantly altered (*p* < 0.10) between HF and LF steers. The affected enzymatic pathways are fatty acyl CoA elongation (*p* = 0.03), linoleic acid transport in via diffusion (*p* = 0.03), fatty acid transport via diffusion (*p* = 0.07), fatty acyl-CoA desaturase (*p* = 0.09), and fatty-acid CoA ligase (*p* = 0.09).

## 4. Discussion

Blood metabolome represents a functional read-out of overall metabolisms in the body; thus, the changes in plasma carboxyl-metabolome observed in this study might have originated from differences in ruminal microbial lipid metabolism and intestinal digestive and absorptive capacity of the animals as well as non-dietary sources such as fatty acid metabolism in the liver, adipose tissue, and muscles [3,16]. The rumen microbiota is known to metabolize lipid and variation in the concentrations of lipid metabolic products in the rumen has been shown to be associated with ADG divergence in beef steers [3]. For instance, ruminal and plasma concentrations of LCFA, including linolenic acid, docosahexaenoic acid, arachidonic, and vaccenic acid were lower in beef steers with greatest ADG compared with those with the least ADG [3].

The intestine plays a significant role, both in digestion and absorption of nutrients such as fatty acids, amino acids, and carbohydrates [17], which implies that differences in the intestinal capacity could consequentially lead to altered plasma metabolome. Previous studies have established relationship between certain intestinal characteristics (such as mucosal density and gene-expression) and feed efficiency. Foote et al. [18] reported altered gene expression in the jejunum mucosa of beef steers with divergent ADG and DMI and concluded that the higher ADG group might have higher potential to digest and absorb nutrients in the small intestine. The authors reported upregulated expression of genes involved in linoleic metabolism (*PLB1* and *CYP3A4*) and arachidonic acid metabolism (*PLB1* and *CYP2B6*) in the jejunum of the high ADG group, which might explain the higher metabolism and relatively lower plasma concentrations of linoleic acid and arachidonic acid as observed for the HF steers in the current study. Furthermore, Spector [19] showed that with increased production of arachidonic acid epoxygenase, a product of CYP2B6 gene, arachidonic acid undergoes increased epoxidation, which yields derivatives that are isomers of epoxyeicosatrienoic acids. This probably explains the greater relative abundance of 11-hydroxy-14,15-epoxy-5Z,8Z,12E-eicosatrienoic acid in the plasma of HF, compared to LF steers observed in the current study. Eicosatrienoic acids have been reported to cause vasodilation, which could result in improved nutrient absorption from small intestine [18,19]. This evidence suggests the possibility that the observed variabilities between HF and LF in the current study could be due to differences in intestinal gene expression, digestion, absorption, and post-digestive metabolism capacity.

Another explanation for the altered plasma carboxyl-metabolome observed in this study is adipose tissue and/or skeletal muscle lipid metabolism. The relative abundance of plasma retinoic acid (a derivative of vitamin A) was greater in LF, relative to HF steers. Retinoic acid has been reported to influence intramuscular fat deposition, being implicated to inhibit adipocyte differentiation, while upregulating adipogenesis-inhibiting genes [20,21,22]. Furthermore, retinoic acid activity has been reported to enhance lipid oxidation pathways in adipocytes [23]. In contrast, beef cattle with lower vitamin A concentration had been reported to have increased intramuscular fat deposition [24]. This evidence is consistent with the result of the current study and might explain the higher plasma LCFA concentrations observed in LF steers, since the observed higher plasma retinoic acid could be antecedent to increased lipid mobilization. The potentially reduced fat deposition in LF steers might explain the lesser ADG for this group.

Another source of variation in the plasma fatty acid profile is hepatic lipid metabolism. Beta-oxidation of fatty acids in the liver cells generates acetyl-CoA, which can either enter the citric acid cycle to generate energy in the form of ATP for hepatic functions [25] or is converted to short-chain oxidative fuels, including ketone bodies and acetate, which are exported via the blood to serve as a source of metabolic energy for several extrahepatic tissues, including skeletal, muscle, and brain tissues during high energy demands to support increased growth [26]. Higher plasma concentrations of short chain fatty acids (such as acetate, butanoic acid, and hydroxyisovalerate) and ketone bodies (such as 3-hydroxybutyrate and 3-isohydroxybutyrate), accompanied with lower plasma concentrations of LCFA probably suggest a greater hepatic beta-oxidation capacity in HF compared to LF steers. This is further confirmed by greater plasma concentrations of leukotriene B4 and 11H-14,15-EETA, two metabolic products of arachidonic acid catabolism in the hepatocytes [27], in HF steers. In addition, benzoic acid and its metabolites can reduce fatty acid biosynthesis in the liver by inhibiting the action of acetyl-CoA carboxylase, an enzyme that catalyzes the ATP-dependent carboxylation of acetyl-CoA to malonyl-CoA, an inhibitor of fatty acid oxidation [28,29]. Since fatty acid biosynthesis and oxidation are reciprocally regulated [30], increased plasma concentration of 4-ethylbenzoic acid in HF steers possibly caused decreased action of acetyl-CoA carboxylase, which probably indicate increased hepatic fatty acid oxidation. In agreement with our results, a similar study reported reduced plasma concentrations of LCFA such as stearic acid, linolenic acid, arachidonic acid, and docosahexaenoic acid in beef steers with high ADG compared to those with low ADG [3].

The results of MSEA of the differentially abundant metabolites revealed that five pathways/enzymes were significantly altered; the affected pathways/enzymes are fatty acyl CoA elongation, linoleic acid transport in via diffusion, fatty acid transport via diffusion, fatty acyl-CoA desaturase, and fatty-acid CoA ligase, indicating the potential roles of these pathways/enzymes in the performance of beef cattle. Fatty acid elongation and fatty acyl-CoA desaturases play a key role in the biosynthesis of polyunsaturated LCFA [31]. Fatty acid elongation is catalyzed by fatty acid elongases, a group of four enzymes (3-keto acyl-CoA synthase, 3-keto acyl-CoA reductase, 3-hydroxy acyl-CoA dehydratase, and trans-2, 3-enoyl-CoA reductase) [32,33]; fatty acyl-CoA desaturases introduce a double bond on the acyl chain of LCFA, and fatty acid transport proteins enhance the transport of LCFA (16–20 carbon atoms) across the mitochondrial and peroxisomal membrane [34]. Once inside the cell, the fatty acids are activated to the corresponding acyl CoA by fatty acyl-CoA ligase in order for oxidation to proceed [35]. Due to the different roles of these enzymatic pathways in different tissues or organs, it is impossible to determine how the regulation of these enzymatic pathways contribute to the altered plasma carboxyl-metabolome between HF and LF steers. In agreement with the results observed in this study, Artegoitia et al. [16] analyzed the lipidomics profile of multiple tissues (duodenum, liver, adipose, and longissimus-dorsi) and identified lipid transport and oxidation as the major lipid metabolic pathways associated with differences in weight gain of beef cattle.

## 5. Conclusions

This study demonstrated that beef steers with divergent ADG had altered plasma carboxyl-metabolome, which is possibly due to differential activities or abundances of enzymes involved in fatty acid catabolism and biosynthesis in several tissues including adipose tissue, duodenum, liver, muscle as well as rumen microbiota. Future research is needed to determine the mechanisms that contribute to alteration in the plasma fatty acid profile of beef cattle using a greater number of animals with a particular focus on the potential roles of fatty acyl CoA elongases, fatty acid transport proteins, fatty acyl-CoA desaturase, and fatty-acid CoA ligase, and how the activities of these enzymes differ between animals with divergent growth performance.

## Figures and Tables

**Table 1:** Differentially abundant plasma carboxylic-acid-containing metabolites between LF and HF steers.

Metabolite	FC	FDR	Identification Level
6-amino-2-oxohexanoate	0.52	0.02	Tier 2
8-methyl-6-nonenoic acid	0.53	0.01	Tier 2
Citronellate	0.53	0.01	Tier 2
12-oxo-9(Z)-dodecenoic acid	0.54	0.01	Tier 2
Oleic acid	0.55	0.03	Tier 1
Isomer 1 of oleic acid	0.58	0.04	Tier 1
Retinoic acid	0.56	0.01	Tier 1
Isomer of retinoic acid	0.57	0.04	Tier 2
Isomer 2 of oleic acid	0.56	0.03	Tier 1
Linoleate	0.56	0.03	Tier 1
9-cis,11-trans-octadecadienoate	0.56	0.03	Tier 2
Octadecatrienoic acid	0.58	0.04	Tier 2
Hexadecenoic acid	0.59	0.04	Tier 2
9Z-hexadecenoic acid	0.59	0.04	Tier 2
Pentadecylic acid	0.59	0.03	Tier 1
Myristic acid	0.60	0.04	Tier 1
Arachidonic acid	0.60	0.03	Tier 1
9,10,18-trihydroxystearate	0.60	0.04	Tier 2
Isomer of pentadecylic acid	0.60	0.04	Tier 1
9,10,18-trihydroxystearate	0.61	0.04	Tier 2
9,10-epoxy-18-hydroxystearate	0.61	0.05	Tier 2
9Z-hexadecenoic acid	0.62	0.04	Tier 2
Octadecatrienoic acid	0.62	0.04	Tier 2
Arachidonic acid	0.62	0.04	Tier 2
Tetradecanoic acid	0.64	0.04	Tier 2
8-methyl-6-nonenoic acid	0.66	0.02	Tier 2
syn-Stemoden-19-oate	0.66	0.02	Tier 2
Eicosatrienoic acid	0.67	0.04	Tier 2
Isomer eicosadienoic acid	0.67	0.04	Tier 2
16-oxopalmitate	0.68	<0.01	Tier 2
Isomer of eicosatrienoic acid	0.69	0.04	Tier 2
18-oxooleate	0.71	0.05	Tier 2
6-hydroxy-5-isopropenyl-2-methylhexanoate	0.82	0.01	Tier 2
3-isohydroxybutyrate	1.22	<0.01	Tier 2
Butanoic acid	1.29	0.05	Tier 2
Hydroxyisovalerate	1.29	0.04	Tier 2
Leukotriene B4	1.33	0.03	Tier 2
Acetate	1.34	0.03	Tier 1
3-hydroxybutyrate	1.35	<0.01	Tier 1
Isomer of acetate	1.42	0.02	Tier 2
11H-14,15-EETA	1.48	0.01	Tier 2
4-ethylbenzoic acid	1.50	0.05	Tier 1
L-threo-3-methylaspartate	1.87	0.01	Tier 2

FC (HF/LF): fold change relative to LF; LF = beef steers with lowest average daily gain; HF = beef steers with highest average daily gain. Only metabolites with both FC ≥ 1.2 or ≤ 0.83, relative to LF, and false discovery rate (FDR) ≤ 0.05 are shown. Tier 1—Positive Identification (CIL Library); Tier 2—High Confidence Putative Identification (Linked Identity Library).

## Data Availability

The data presented in this study are available on request from the corresponding author.

## Supplementary Materials

The following are available online at https://www.mdpi.com/2076-2615/11/1/67/s1, Table S1: List of identified metabolites.

## References

[1] Gonano C.V., Montanholi Y.R., Schenkel F.S., Smith B.A., Cant J.P., Miller S.P. (2014). The relationship between feed efficiency and the circadian profile of blood plasma analytes measured in beef heifers at different physiological stages. Animal.

[2] Alexandre P.A., Kogelman L.J.A., Santana M.H.A., Passarelli D., Pulz L.H., Fantinato-Neto P., Silva P.L., Leme P.R., Strefezzi R.F., Coutinho L.L. (2015). Liver transcriptomic networks reveal main biological processes associated with feed efficiency in beef cattle. BMC Genom..

[3] Artegoitia V.M., Foote A.P., Lewis R.M., Freetly H.C. (2017). Rumen Fluid Metabolomics Analysis Associated with Feed Efficiency on Crossbred Steers. Sci. Rep..

[4] Fonseca L.D., Eler J.P., Pereira M.A., Rosa A.F., Alexandre P.A., Moncau C.T., Salvato F., Rosa-Fernandes L., Palmisano G., Ferraz J.B.S. (2019). Liver proteomics unravel the metabolic pathways related to Feed Efficiency in beef cattle. Sci. Rep..

[5] Psychogios N., Hau D.D., Peng J., Guo A.C., Mandal R., Bouatra S., Sinelnikov I., Krishnamurthy R., Eisner R., Gautam B. (2011). The Human Serum Metabolome. PLoS ONE.

[6] Goldansaz S.A., Guo A.C., Sajed T., Steele M.A., Plastow G.S., Wishart D.S. (2017). Livestock metabolomics and the livestock metabolome: A systematic review. PLoS ONE.

[7] Zhao S., Li H., Han W., Chan W., Li L. (2019). Metabolomic Coverage of Chemical-Group-Submetabolome Analysis: Group Classification and Four-Channel Chemical Isotope Labeling LC-MS. Anal. Chem..

[8] Ogunade I.M., McCoun M. (2020). Average daily gain divergence in beef steers is associated with altered plasma metabolome and whole blood immune-related gene expression. Transl. Anim. Sci..

[9] Mukiibi R., Vinsky M., Keogh K.A., Fitzsimmons C., Stothard P., Waters S.M., Li C. (2018). Transcriptome analyses reveal reduced hepatic lipid synthesis and accumulation in more feed efficient beef cattle. Sci. Rep..

[10] Guo K., Li L. (2010). High-Performance Isotope Labeling for Profiling Carboxylic Acid-Containing Metabolites in Biofluids by Mass Spectrometry. Anal. Chem..

[11] Wu Y., Li L. (2012). Determination of Total Concentration of Chemically Labeled Metabolites as a Means of Metabolome Sample Normalization and Sample Loading Optimization in Mass Spectrometry-Based Metabolomics. Anal. Chem..

[12] Mung D., Li L. (2017). Development of Chemical Isotope Labeling LC-MS for Milk Metabolomics: Comprehensive and Quantitative Profiling of the Amine/Phenol Submetabolome. Anal. Chem..

[13] Huan T., Li L. (2015). Quantitative Metabolome Analysis Based on Chromatographic Peak Reconstruction in Chemical Isotope Labeling Liquid Chromatography Mass Spectrometry. Anal. Chem..

[14] Li L., Li R., Zhou J., Zuniga A., Stanislaus A.E., Wu Y., Huan T., Zheng J., Shi Y., Wishart D.S. (2013). MyCompoundID: Using an Evidence-Based Metabolome Library for Metabolite Identification. Anal. Chem..

[15] Xia J., Broadhurst D.I., Wilson M., Wishart D.S. (2013). Translational biomarker discovery in clinical metabolomics: An introductory tutorial. Metabolomics.

[16] Artegoitia V.M., Foote A.P., Lewis R.M., Freetly H.C. (2019). Metabolomics Profile and Targeted Lipidomics in Multiple Tissues Associated with Feed Efficiency in Beef Steers. ACS Omega.

[17] Lindholm-Perry A.K., Butler A.R., Kern R.J., Hill R., Kuehn L.A., Wells J.E., Oliver W.T., Hales K.E., Foote A.P., Freetly H.C. (2016). Differential gene expression in the duodenum, jejunum and ileum among crossbred beef steers with divergent gain and feed intake phenotypes. Anim. Genet..

[18] Foote A.P., Keel B.N., Zarek C.M., Lindholm-Perry A.K. (2017). Beef steers with average dry matter intake and divergent average daily gain have altered gene expression in the jejunum. J. Anim. Sci..

[19] Spector A.A. (2009). Arachidonic acid cytochrome P450 epoxygenase pathway. J. Lipid Res..

[20] Bonet M.L., Ribot J., Felipe F., Palou A. (2003). Vitamin A and the regulation of fat reserves. Cell. Mol. Life Sci..

[21] Lindholm-Perry A.K., Cunningham H.C., Kuehn L.A., Vallet J.L., Keele J.W., Foote A.P., Cammack K.M., Freetly H.C. (2017). Relationships between the genes expressed in the mesenteric adipose tissue of beef cattle and feed intake and gain. Anim. Genet..

[22] Sato M., Hiragun A., Mitsui H. (1980). Preadipocytes possess cellular retinoid binding proteins and their differentiation is inhibited by retinoids. Biochem. Biophys. Res. Commun..

[23] Berry D.C., DeSantis D., Soltanian H., Croniger C.M., Noy N. (2012). Retinoic Acid Upregulates Preadipocyte Genes to Block Adipogenesis and Suppress Diet-Induced Obesity. Diabetes.

[24] Oka A., Maruo Y., Miki T., Yamasaki T., Saito T. (1998). Influence of vitamin A on the quality of beef from the Tajima strain of Japanese Black cattle. Meat Sci..

[25] Allen M.S. (2014). Drives and limits to feed intake in ruminants. Anim. Prod. Sci..

[26] Rui L. (2014). Energy Metabolism in the Liver. Compr. Physiol..

[27] Hanna V.S., Hafez E.A.A. (2018). Synopsis of arachidonic acid metabolism: A review. J. Adv. Res..

[28] Ohmori K., Yamada H., Yasuda A., Yamamoto A., Matsuura N., Kiniwa M. (2003). Anti-hyperlipidemic action of a newly synthesized benzoic acid derivative, S-2E. Eur. J. Pharmacol..

[29] Tong L., Harwood H.J. (2006). Acetyl-coenzyme A carboxylases: Versatile targets for drug discovery. J. Cell. Biochem..

[30] Foster D.W. (2012). Malonyl-CoA: The regulator of fatty acid synthesis and oxidation. J. Clin. Investig..

[31] Lee J.M., Lee H., Kang S., Park W.J. (2016). Fatty Acid Desaturases, Polyunsaturated Fatty Acid Regulation, and Biotechnological Advances. Nutrients.

[32] Cinti D.L., Cook L., Nagi M.N., Suneja S.K. (1992). The fatty acid chain elongation system of mammalian endoplasmic reticulum. Prog. Lipid Res..

[33] Jakobsson A., Westerberg R., Jacobsson A. (2006). Fatty acid elongases in mammals: Their regulation and roles in metabolism. Prog. Lipid Res..

[34] Anderson C.M., Stahl A. (2013). SLC27 fatty acid transport proteins. Mol. Asp. Med..

[35] Londesborough J.C., Webster L.T., Boyer P.D. (1974). 14. Fatty Acyl-CoA Synthetases. The Enzymes.

